# Colibactin (*pks*) carriage in *Escherichia coli* is associated with lineage restriction, reduced plasmid burden, and lower antimicrobial resistance

**DOI:** 10.3389/fmicb.2026.1842853

**Published:** 2026-05-26

**Authors:** Adel Azour, Ghassan M. Matar, Melhem Bilen

**Affiliations:** 1 Department of Experimental Pathology, Immunology, and Microbiology, Faculty of Medicine, American University of Beirut, Beirut, Lebanon; 2 Centre of Infectious Diseases Research, American University of Beirut, Beirut, Lebanon; 3 World Health Organization (WHO) Collaborating Centre for Reference and Research on Bacterial Pathogens, Beirut, Lebanon

**Keywords:** antimicrobial resistance, colibactin, *Escherichia coli*, pangenome dynamics, population genomics

## Abstract

The colibactin *pks* genomic island of *Escherichia coli* is a chromosomally integrated secondary metabolite locus implicated in host–microbe interactions and colorectal carcinogenesis. Although *pks* distribution has been linked to specific *E. coli* lineages, its relationship with plasmid burden, antimicrobial resistance (AMR), and genome-wide accessory gene structure remains incompletely resolved at population scale. Here, we analyzed 9,700 curated *E. coli* genomes from BV-BRC and identified 498 genomes carrying complete or near-complete canonical *pks* loci. *pks*-positive genomes were strongly enriched in phylogroup B2 and concentrated within a limited number of sequence types, including ST73, ST95, ST127, and ST12. Compared with *pks-*negative genomes, *pks*-positive genomes carried fewer AMR genes and fewer plasmid replicons. This pattern persisted in phylogroup B2-controlled comparisons, although sequence-type structure remained an important potential contributor. Synteny and flanking-region analyses showed strong conservation of the internal *clb* gene cluster together with recurrent conservation of the surrounding chromosomal neighborhood, consistent with positional stability of canonical *pks* loci. Pangenome reconstruction of B2-matched genomes revealed that *pks*-positive genomes had a smaller total pangenome and accessory gene compartment, but a larger core genome, than *pks-*negative genomes. Together, these findings indicate that canonical *pks* carriage marks a lineage-restricted and genomically conserved *E. coli* subpopulation associated with reduced plasmid and AMR burden. These associations support a model in which colibactin-positive lineages differ from multidrug-resistant plasmid-rich lineages in their accessory genome architecture, although experimental studies remain required to define the underlying mechanisms.

## 1 Introduction

*Escherichia coli* is a highly diverse bacterial species occupying a broad range of ecological niches, from commensal colonization of the human gut to extraintestinal pathogenic lifestyles associated with severe infections (Kaper et al., 2004). This remarkable ecological versatility is largely driven by horizontal gene transfer, which enables the rapid acquisition of accessory genetic elements encoding antimicrobial resistance (AMR), virulence factors, and metabolic traits. While plasmids represent the primary vectors of AMR dissemination in *E. coli*, chromosomally encoded pathogenicity islands can mediate long-term stabilization of virulence functions within specific lineages (Partridge et al., 2018).

One of the most striking examples of such chromosomally embedded virulence determinants is the colibactin biosynthetic gene cluster (*pks* island). This 54-kb genomic island encodes a non-ribosomal peptide–polyketide hybrid pathway responsible for the synthesis of colibactin, a genotoxin that induces DNA double-strand breaks, chromosomal instability, and mutational signatures linked to colorectal cancer (Faïs et al., 2018; Healy et al., 2016; Lv et al., 2025; Nougayrède et al., 2006; Taieb et al., 2016). Experimental and epidemiological studies have implicated *pks*-positive *E. coli* strains in host–microbiome interactions with long-term pathological consequences. Although the phylogenetic distribution, chromosomal integration, and evolutionary dynamics of the *pks* island have been increasingly characterized (Faïs et al., 2018; Putze et al., 2009; Raisch et al., 2014; Suresh et al., 2021), the relationship between canonical *pks* carriage, plasmid burden, antimicrobial resistance content, and genome-wide accessory gene structure remains insufficiently resolved at large population scale.

Previous studies have reported enrichment of *pks* among phylogroup B2 lineages, particularly within extraintestinal pathogenic *E. coli* (ExPEC), although many analyses were based on smaller or geographically restricted collections (Faïs et al., 2018; Suresh et al., 2021). Although predominant chromosomal maintenance is supported in several B2-associated lineages, the relative contribution of historical mobility, lineage stabilization, and contemporary accessory genome interactions remains incompletely resolved. Moreover, the relationship between *pks* carriage and antimicrobial resistance burden has not been systematically investigated at global population scale, despite growing evidence that virulence and resistance traits may follow distinct evolutionary trajectories.

We hypothesized that if canonical *pks* loci are predominantly maintained through long-term vertical inheritance, *pks*-positive *E. coli* would be concentrated within specific lineages and display genomic features associated with reduced plasmid burden, lower AMR content, and greater chromosomal conservation relative to *pks*-negative strains.

Here, we performed a global population-genomic analysis of the *pks* island across 9,700 *E. coli* genomes to test associations between canonical *pks* carriage, lineage structure, antimicrobial resistance burden, plasmid content, and chromosomal conservation.

## 2 Materials and methods

### 2.1 Genome dataset construction and quality control

A total of 12,053 *E. coli* genomes were retrieved from the BV-BRC database (https://www.bv-brc.org/, accessed 5 August 2025). Species identity and taxonomic consistency were verified using FastANI v1.33 (Jain et al., 2018), and only genomes showing ≥95% average nucleotide identity to the *E. coli* type strain were retained. Assemblies were filtered based on quality metrics including genome size (4.5–5.5 Mb), GC content (50%−51.5%), contig number ( ≤ 100), and removal of duplicate assemblies. After quality control, a curated dataset of 9,700 high-quality genomes was retained for downstream analyses.

### 2.2 *pks* island detection and validation

The presence of the colibactin (*pks*) island was screened using BLASTN searches against the reference *pks* locus from strain IHE3034 (GenBank accession AM229678.1), with hits retained at ≥80% nucleotide identity and ≥50 kb alignment length. The primary *pks* screen was intentionally conservative and designed to identify high-confidence complete or near-complete canonical *pks* loci. Consequently, fragmented loci in draft assemblies or divergent *pks*-like regions may have been under-detected. For each positive genome, the contig harboring the *pks* locus was extracted from the original FASTA assemblies and annotated using Prokka v1.14.6, supplemented with curated colibactin protein references to ensure accurate identification of *clb* genes. Annotation completeness was verified by confirming the presence of the full *clbA–clbS* gene set using BLASTP validation (≥90% amino acid identity and ≥90% query coverage).

### 2.3 ARG and plasmid replicon annotation

Antimicrobial resistance genes (ARGs) were identified using ResFinder (identity ≥90%, coverage ≥80%; Center for Genomic Epidemiology, n.d.), and plasmid replicons were detected using PlasmidFinder (identity ≥95%, coverage ≥80%; Carattoli et al., 2014), ARG–plasmid associations were assessed using a conservative contig-level co-occurrence approach, in which ARGs located on the same contig as a plasmid replicon marker were classified as replicon-associated. Because most public assemblies are fragmented draft genomes, this approach was not used to make definitive claims regarding plasmid localization and may underestimate plasmid-borne ARGs.

### 2.4 Phylogroup, MLST, and metadata analysis

Phylogroups were assigned using EzClermont (Clermont et al., 2013), and sequence types (STs) were determined by MLST (Wirth et al., 2006). Host origin and geographic metadata were extracted from BV-BRC records.

### 2.5 Synteny and flanking-region analysis

To evaluate structural conservation of the *pks* island, an initial set of complete *pks*-containing contigs was screened, from which six representative genomes were selected for synteny visualization based on complete *clbA*–*clbS* representation, contig continuity across the *pks* locus, host-origin diversity, sequence-type diversity, and figure readability. Gene cluster synteny analysis was then performed using clinker, with interactive visualization generated *via* clustermap.js. To assess positional conservation of the *pks* island, the chromosomal context of *pks*-containing contigs was examined using Prokka-generated GFF annotations. Genes immediately upstream and downstream of the *clb* cluster were extracted, and presence/absence matrices of flanking genes were constructed across genomes. Features present in ≥5% of genomes were retained, and binary heatmaps of upstream and downstream flanking regions were generated in Python to evaluate conservation of the *pks* chromosomal neighborhood.

### 2.6 Pangenome reconstruction

Pangenome reconstruction of B2-matched *pks*-positive and *pks*-negative genomes was performed using Panaroo under strict mode to minimize annotation artifacts.

### 2.7 Statistical analysis

Statistical analyses were conducted in R (RStudio 2023.12.03 + 69 “Ocean Storm”) and Python (version 3.12.3). Differences in ARG counts between *pks*-positive and *pks*-negative genomes were assessed using the Wilcoxon rank-sum test, associations between *pks* carriage and phylogroup or ST distribution were evaluated using Fisher's exact tests, and pangenome size comparisons were assessed using permutation tests (10,000 iterations). *P*-values were adjusted for multiple testing using the Benjamini–Hochberg false discovery rate correction. Data visualization was performed using ggplot2 and seaborn.

## 3 Results

### 3.1 Prevalence and population structure of *pks*-positive *E. coli* genomes

**Figure 1:**
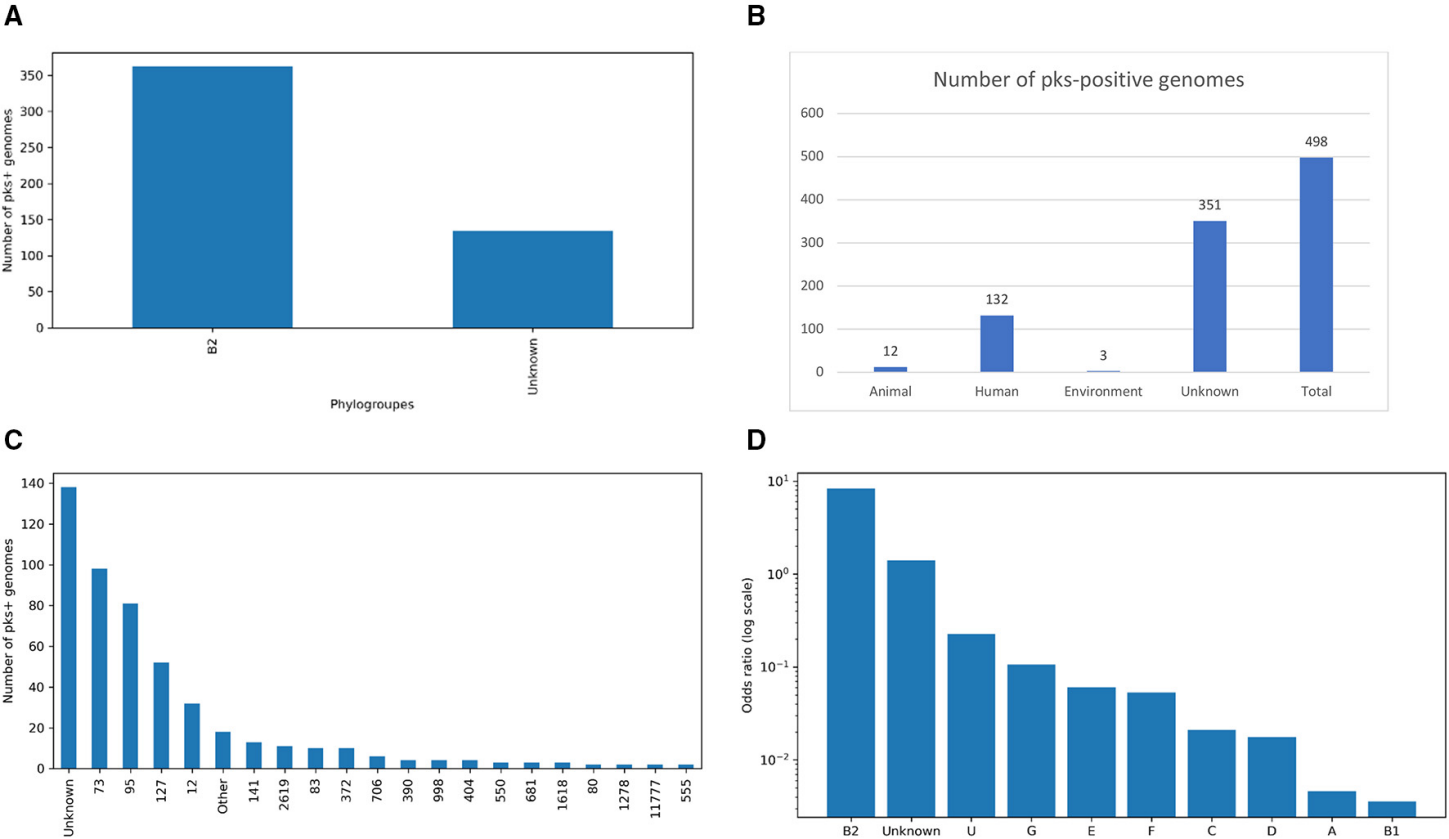
**(A)** Phylogroup distribution of *pks*-positive *E. coli* genomes. Bar plot showing the number of *pks*-positive *E. coli* genomes assigned to each phylogroup. The majority of *pks*-positive genomes belong to phylogroup B2, while a substantial fraction is classified as phylogroup unknown due to missing or ambiguous phylogroup annotations. (**B)** Host distribution of *pks*-positive *E. coli* genomes. Bar plot showing the number of *pks*-positive *E. coli* genomes according to host origin. Genomes are classified as human-associated, animal-associated, environment-associated, or unknown when host metadata were unavailable. The total number of *pks*-positive genomes analyzed (*n* = 498) is shown for reference. **(C)** Sequence type distribution of *pks*-positive *E. coli* genomes. Bar plot showing the number of *pks*-positive *E. coli* genomes assigned to each multilocus sequence type (ST). The most prevalent sequence types are shown individually, while low-frequency STs are grouped as “Other”. Genomes lacking ST assignment are classified as “Unknown”. (**D)** Phylogroup enrichment of *pks*-positive *E. coli* genomes. Odds ratios (log10 scale) were calculated to assess the enrichment or depletion of *pks*-positive genomes across *E. coli* phylogroups relative to the full genome collection. Values greater than 1 indicate phylogroups enriched in *pks*-positive genomes, whereas values below 1 indicate phylogroups depleted in *pks*-positive genomes. Although *pks*-positive genomes were predominantly assigned to phylogroup B2, odds ratio analysis highlights strong enrichment of *pks* within B2 and marked depletion across all other phylogroups. Statistical significance of enrichment and depletion was assessed using Fisher's exact test (B2 enrichment, *P* < 0.001).

Among the full *E. coli* genome collection analyzed, 498 genomes were identified as *pks*-positive, corresponding to strains harboring a complete or near-complete colibactin biosynthetic gene cluster. Phylogroup assignment revealed a strong population structure, with *pks*-positive genomes associated with phylogroup B2, which accounted for approximately 73% (362/498) of all *pks*-positive genomes (Figure 1A). The remaining genomes were classified as phylogroup unknown (136/498), while no substantial representation was observed for phylogroups A, B1, C, D, E, F, or G.

### 3.2 Host-origin distribution of *pks*-positive genomes

Host metadata analysis indicated that human-associated isolates represented the largest annotated category among *pks*-positive genomes in raw counts (132 genomes), followed by animal-associated isolates (12 genomes), while environmental isolates were rare (3 genomes). A large proportion of genomes lacked explicit host metadata (347 genomes classified as unknown; Figure 1B). However, enrichment analysis relative to the full dataset showed that human-associated isolates were not overrepresented among pks-positive genomes (OR = 0.65, FDR-adjusted *P* < 0.001), whereas animal-associated isolates were significantly depleted (OR = 0.09, FDR-adjusted *P* < 0.001). Interpretation remains limited by the large proportion of genomes lacking host annotation (Supplementary Figure S1).

### 3.3 *pks* carriage is concentrated within a limited set of sequence types

Sequence type (ST) profiling demonstrated that *pks* carriage was concentrated within a limited number of clonal backgrounds (Figure 1C). The most frequent STs among *pks*-positive genomes were ST73 (*n* = 98), ST95 (*n* = 81), ST127 (*n* = 52), and ST12 (*n* = 32), together accounting for approximately 53% of all *pks*-positive genomes. Additional STs, including ST141, ST2619, ST83, ST372, and ST706, were detected at lower frequencies (each *n* ≤ 15), while the remaining genomes were distributed across numerous low-abundance STs.

Odds ratio analysis confirmed a strong enrichment of *pks* within phylogroup B2, with odds ratios approaching 10-fold enrichment of *pks* in B2 (Figure 1D). In contrast, phylogroups A and B1 exhibited marked depletion, with odds ratios below 0.01. Sequence-type enrichment analysis relative to the full dataset further confirmed that pks carriage was significantly overrepresented in ST73, ST95, ST127, and ST12, whereas multidrug-resistant ST131 was comparatively depleted (Supplementary Figure S2). These findings indicate that the observed ST distribution reflects selective lineage enrichment rather than raw database composition alone.

### 3.4 ARG burden in *pks*-positive vs. *pks*-negative genomes

**Figure 2:**
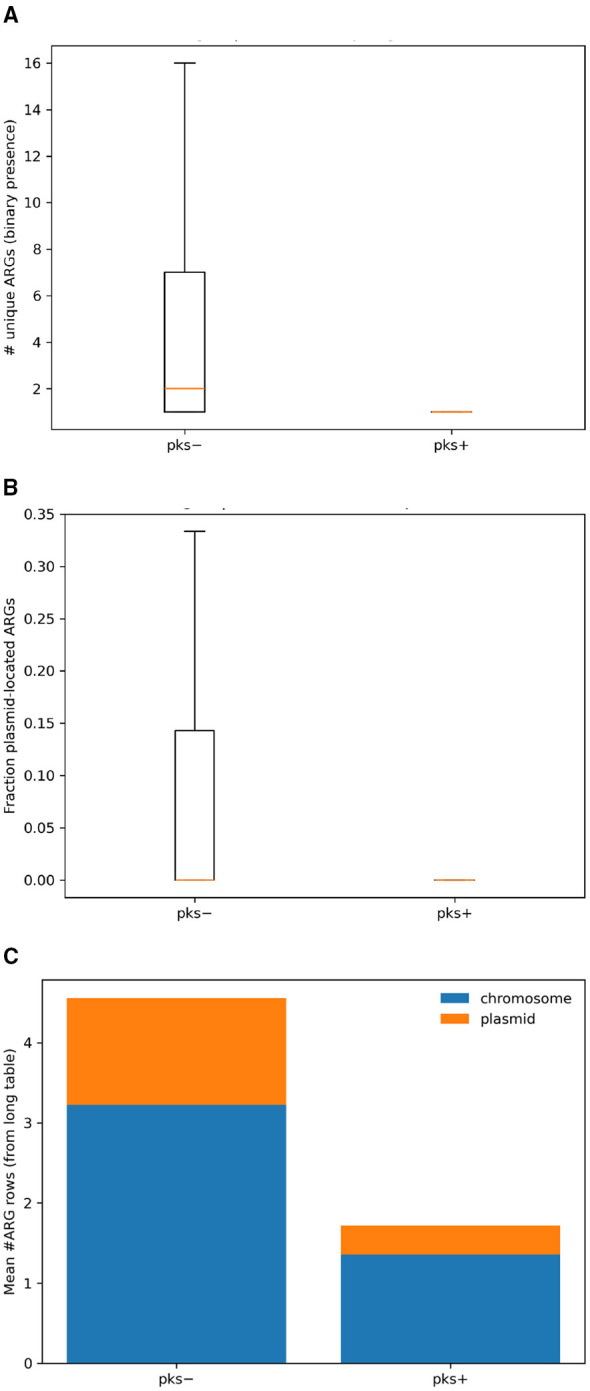
**(A)** ARG burden in *pks*-positive and *pks*-negative *E. coli* genomes. Boxplots showing the distribution of the number of unique ARGs detected per genome in *pks*-negative and *pks*-positive *E. coli* populations. **(B)** Fraction of plasmid-associated ARGs in *pks*-positive and *pks*-negative *E. coli* genomes. Boxplots showing the fraction of ARGs located on plasmids relative to the total ARG content per genome in *pks*-negative and *pks*-positive *E. coli* populations. For each genome, the plasmid-associated ARG fraction was calculated as the number of plasmid-located ARGs divided by the total number of detected ARGs. Boxes represent the interquartile range, the central line indicates the median, and whiskers denote the full range of observed values. **(C)** Genomic localization of ARGs in *pks*-positive and *pks*-negative *E. coli* genomes. Stacked bar plots showing the mean number of ARGs per genome localized on the chromosome or on plasmids in *pks*-negative and *pks*-positive *E. coli* populations. Mean values were calculated across all genomes within each group, highlighting differences in chromosomal and plasmid-associated ARG content between *pks*-positive and *pks*-negative genomes.

Quantification of ARGs revealed pronounced differences between *pks*-positive and *pks*-negative populations (Figure 2A). *pks*-negative genomes displayed a broad distribution of ARG counts, with a median of 2 ARGs per genome and an upper range extending to 16 or more unique ARGs. In contrast, *pks*-positive genomes exhibited a markedly constrained ARG profile, with a median of 1 ARG per genome and an interquartile range largely restricted between 0 and 1 ARG.

More than 65% of *pks*-positive genomes carried no detectable ARGs, while genomes harboring more than two ARGs were rare (< 5%). By comparison, *pks*-negative genomes frequently carried ≥5 ARGs, with a substantial fraction exceeding 10 ARGs per genome. The difference in ARG burden between *pks*-positive and *pks*-negative genomes was statistically significant (Wilcoxon rank-sum test, *P* < 0.001).

To control for phylogenetic background, we repeated the analysis within phylogroup B2 using matched sets of *pks*-positive and *pks*-negative genomes (Supplementary Figure S3). The same pattern was observed: B2 *pks*-positive genomes carried significantly fewer ARGs than B2 *pks*-negative genomes (Wilcoxon rank-sum test, *P* < 0.001), indicating that the reduced resistance burden of *pks*-positive strains is not solely attributable to phylogroup composition, although lineage structure may also contribute. Because phylogroup B2 contains both *pks*-enriched lineages such as ST73, ST95, ST127, and ST12 and major multidrug-resistant clones such as ST131, we further considered sequence-type structure as a potential confounder. Therefore, the reduced ARG burden observed among *pks*-positive genomes should be interpreted as a lineage-associated population pattern rather than evidence that *pks* carriage alone directly limits AMR acquisition.

### 3.5 Contig-level association between ARGs and plasmid replicon markers in *pks*-positive and *pks*-negative genomes

Analysis of contig-level associations between ARGs and plasmid replicon markers further distinguished *pks*-positive from *pks*-negative genomes (Figures 2B, C). In *pks*-negative genomes, the mean number of ARGs not co-occurring with replicon markers was approximately 3.2 per genome, while ARGs co-occurring with plasmid replicon markers on the same contig contributed an additional mean of 1.3 ARGs per genome.

In contrast, *pks*-positive genomes displayed a mean ARG count of 1.3 not co-occurring with replicon markers and a mean ARG count below 0.4 co-occurring with plasmid replicon markers on the same contig. The majority of *pks*-positive genomes carried no ARGs co-occurring with plasmid replicon markers, with such associations detected only in a small subset of genomes. Consistently, the fraction of ARGs located on plasmids was close to zero in *pks*-positive genomes (median = 0), whereas *pks*-negative genomes showed a wide distribution, with plasmid fractions ranging from 0 to > 0.30.

A phylogroup-controlled comparison within phylogroup B2 further showed that ARG counts co-occurring with plasmid replicon markers tended to be lower in *pks*-positive genomes (Supplementary Figure S4), although this difference did not reach statistical significance (*P* = 0.065).

### 3.6 Plasmid replicon content in *pks*-positive genomes

**Figure 3:**
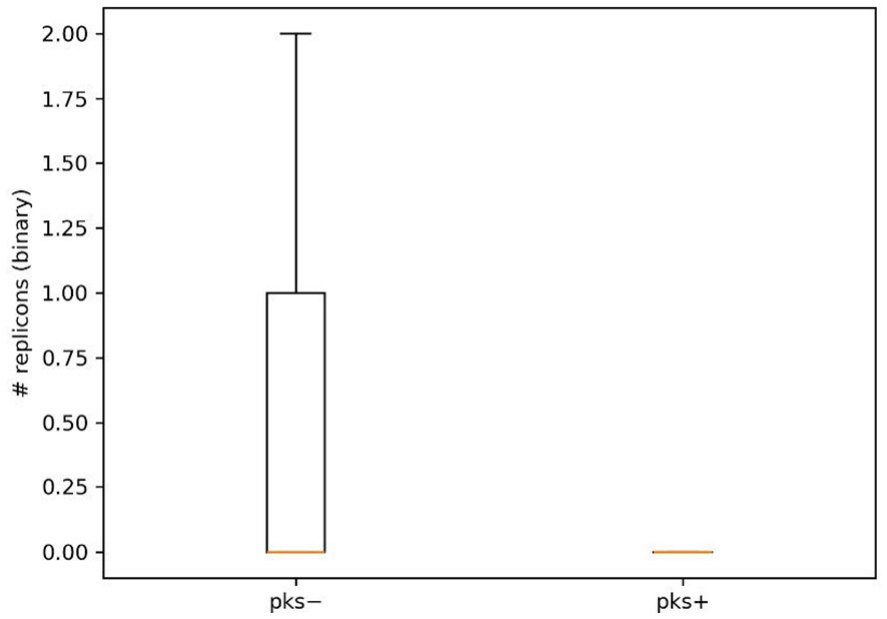
Plasmid replicon richness in *pks*-positive and *pks*-negative *E. coli* genomes. Boxplots showing the distribution of the number of plasmid replicons detected per genome in *pks*-negative and *pks*-positive *E. coli* populations. Plasmid replicons were identified using PlasmidFinder, and replicon presence was treated as a binary variable per genome. Boxes represent the interquartile range, the central line indicates the median, and whiskers denote the full range of observed values.

Plasmid replicon screening revealed marked differences in plasmid carriage between *pks-*positive and *pks*-negative genomes (Figure 3). Among pks-positive genomes (*n* = 498), 85.3% (425/498) lacked detectable plasmid replicons, and genomes carrying more than one replicon were exceedingly rare (0.2%, 1/498).

In contrast, *pks*-negative genomes (*n* = 9,203) exhibited substantially higher plasmid carriage, with 29.2% (2,691/9,203) carrying at least one replicon, and 7.2% (662/9,203) harboring more than one replicon.

### 3.7 Conservation of the *pks* genomic island gene content

To assess gene-level conservation within the *pks* locus, presence–absence profiling of the 19 colibactin-associated genes (*clbA*–*clbS*) was performed across *pks*-positive genomes. A presence–absence heatmap generated from a uniform subsample of 250 genomes demonstrated near-complete conservation of the *pks* gene repertoire (Supplementary Figure S5).

All core biosynthetic genes were detected in more than 95% of genomes, with only sporadic absences affecting individual genes. These missing genes occurred at low frequency and were not restricted to specific phylogroups or sequence types.

Importantly, all *pks*-positive genomes harbored a single, full-length *pks* locus with 100% sequence coverage and extremely high nucleotide identity (median identity >99.9%), with no evidence of partial clusters, truncations, or internal deletions.

### 3.8 Structural conservation of the *pks* locus

**Figure 4:**
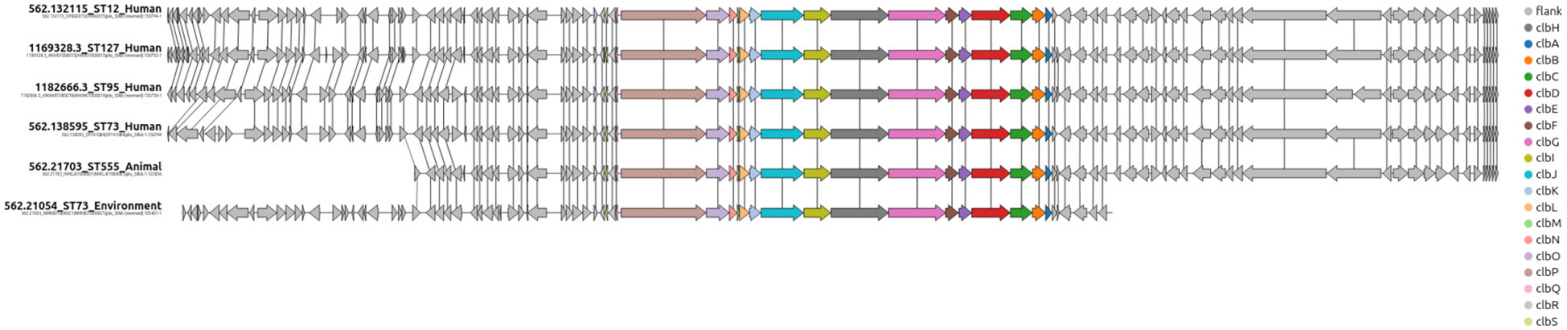
Conservation of the *pks* island architecture across human, animal, and environmental *E. coli* isolates. Comparative synteny of representative pks-positive genomes showing strong internal conservation of the clb cluster and recurrent conservation of the immediate upstream chromosomal neighborhood. Limited variability was mainly restricted to downstream or distal adjacent regions.

Comparative synteny analysis of the *pks* genomic island was conducted across six *E. coli* genomes originating from human, animal, and environmental sources and belonging to multiple sequence types, including ST12, ST127, ST95, ST73, and ST55 (Figure 4). This analysis enabled direct comparison of *pks* island organization across diverse ecological and phylogenetic backgrounds.

Across all six genomes, the *pks* island exhibited a highly conserved internal structure, with the complete *clb* gene cluster (*clbA* to *clbS*) present in all isolates. Gene order and orientation within the cluster were preserved, and alignment links indicated high sequence similarity across homologous *clb* genes. Core biosynthetic components (*clbA–clbN*), transport- and processing-associated genes (*clbM–clbP*), as well as the self-resistance determinant *clbS*, were consistently detected, indicating the absence of major deletions or rearrangements within the *pks* island.

In contrast, the immediate upstream chromosomal neighborhood showed recurrent conservation across genomes, whereas limited variability was observed mainly in downstream or more distal adjacent regions. Differences in local synteny and structural continuity were therefore concentrated outside the core conserved insertion neighborhood, consistent with local microvariation rather than independent insertion at unrelated chromosomal sites.

Notably, conservation of the *pks* island was observed irrespective of isolate origin. Human-derived, animal-derived, and environmental isolates displayed comparable *clb* gene organization and sequence similarity, with no host-specific structural disruptions detected within the cluster. This indicates that the *pks* island is maintained as a conserved genomic module across distinct ecological niches.

Overall, these results demonstrate that the pks island constitutes a highly conserved genetic element within *E. coli*, embedded within a broadly conserved chromosomal neighborhood with limited local variability, supporting its stable maintenance across diverse lineages and host environments.

### 3.9 Pangenome structure of B2-matched *pks*-positive vs. *pks*-negative genomes

To investigate whether carriage of the *pks* island is associated with distinct patterns of genome conservation and diversity within phylogroup B2, we reconstructed the pangenomes of matched sets of *pks*-positive (*n* = 362) and *pks*-negative (*n* = 362) genomes using Panaroo.

**Figure 5:**
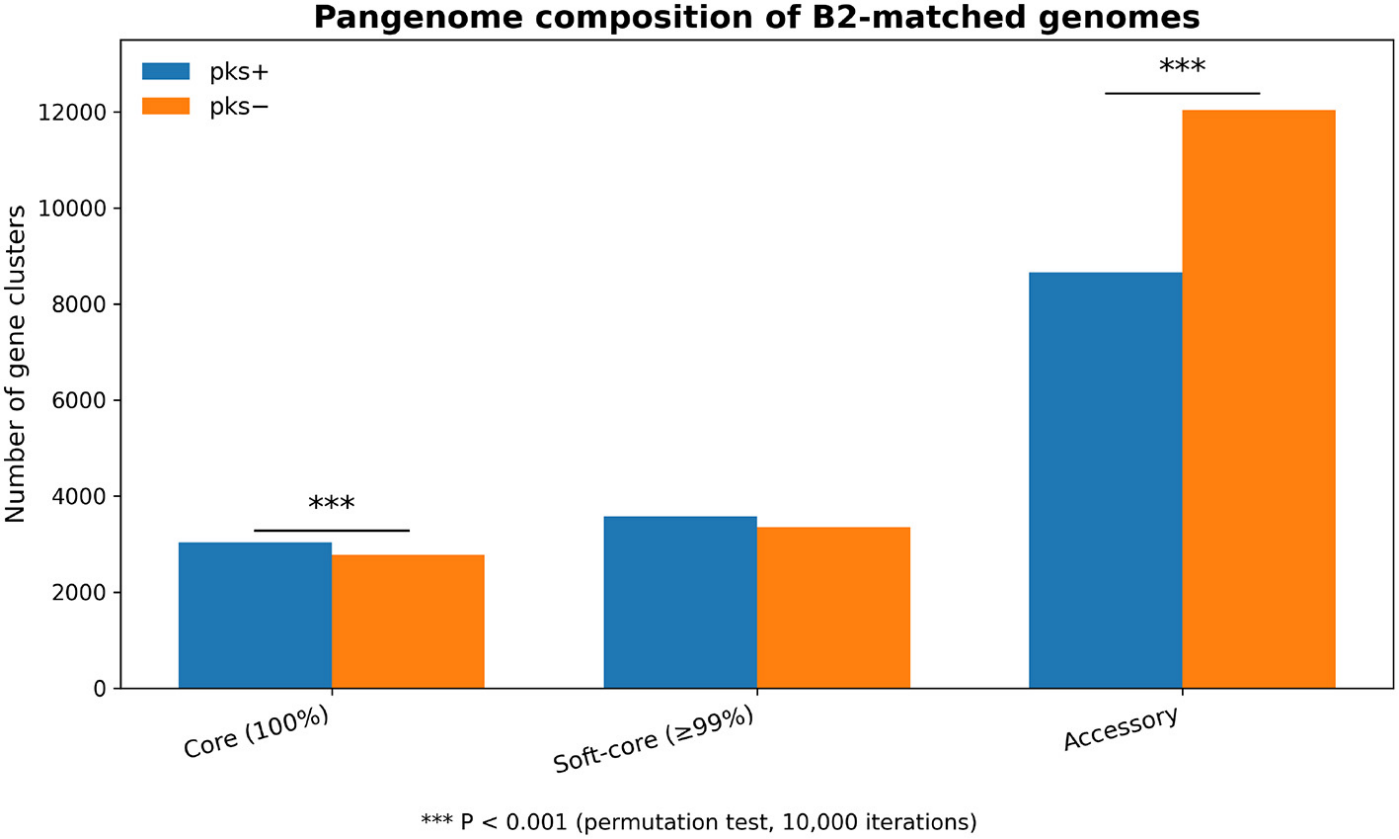
Pangenome composition of B2-matched *pks-*positive and *pks*-negative *E. coli* genomes. Bar plot showing the number of gene clusters in the strict core (100% presence), soft-core (≥99% presence), and accessory genome compartments reconstructed using Panaroo. B2-matched *pks*-positive genomes (blue, *n* = 362) displayed larger core and soft-core compartments, whereas *pks*-negative genomes (orange, *n* = 362) exhibited a markedly expanded accessory genome. ***P < 0.001 (permutation test, 10,000 iterations).

The *pks*-positive group exhibited a total pangenome of 12,238 gene clusters, including 3,039 strict core genes shared by all genomes and 3,580 soft-core genes present in ≥99% of genomes (Figure 5). In contrast, the *pks*-negative group displayed a substantially larger pangenome comprising 15,394 gene clusters, but a smaller strict core (2,777 genes) and soft-core genome (3,357 genes). Consequently, the accessory gene compartment was markedly expanded in *pks*-negative genomes (12,037 genes) compared to *pks*-positive genomes (8,658 genes).

Pangenome accumulation curves further supported this pattern, with the *pks*-negative pangenome expanding more rapidly and reaching higher gene counts as additional genomes were sampled, while the *pks*-positive group maintained a larger core genome across sampling depths (Supplementary Figure S6). Consistently, gene frequency spectrum analyses showed a greater proportion of low-frequency (rare) genes in *pks*-negative genomes, indicative of increased genome plasticity (Supplementary Figure S7). Thus, *pks*-positive B2 lineages have a smaller total pangenome (by ~20%) and a larger core genome than their *pks*-negative counterparts, indicating they are more genomically conserved as a group. Permutation testing (10,000 iterations) confirmed that the larger total pangenome and smaller strict core genome observed in the *pks*-negative comparator set were statistically significant (*P* < 0.001 for total pangenome size difference; *P* < 0.001 for strict core genome difference).

### 3.10 Chromosomal flanking gene conservation surrounding the *pks* locus

To further investigate the chromosomal context of the *pks* island, we constructed a binary presence/absence heatmap of genes flanking the locus across *pks*-positive genomes (Supplementary Figure S8). Flanking regions were defined as the annotated genes immediately upstream and downstream of the *pks* cluster, and features present in ≥5% of genomes were retained for visualization. The resulting heatmap revealed a pronounced block of conserved upstream flanking genes shared by the majority of genomes whereas downstream regions exhibited relatively greater local variability but still contained recurrent conserved elements.

This pattern indicates that the *pks* island is not randomly distributed across the chromosome but instead occupies a consistent genomic neighborhood. The conserved upstream gene block suggests positional stability of the insertion locus, while the limited variation observed downstream likely reflects local micro-rearrangements rather than independent insertion events. Importantly, no evidence was observed for alternative chromosomal integration sites or plasmid-associated contexts, consistent with the hypothesis that the *pks* island represents a long-term chromosomally conserved genomic island likely maintained predominantly through vertical inheritance. This pattern is consistent with previously described integration of the *pks* island near conserved asn tRNA-associated chromosomal loci.

## 4 Discussion

Our analysis shows that *pks*-positive *E. coli* are concentrated within specific phylogenetic and clonal backgrounds and form a lineage-restricted subpopulation associated with chromosomal *pks* integration and reduced markers of horizontal gene acquisition.

By integrating population structure, resistome, plasmid content, and locus conservation across ~500 *pks*-positive genomes we showed a strong and consistent association between *pks* carriage and phylogroup B2, with additional concentration within a limited number of sequence types, notably ST73, ST95, ST127, and ST12. These lineages are well recognized for their role in ExPEC infections and for their enhanced capacity for long-term colonization of the human gut (Johnson et al., 2008; Kaper et al., 2004). Notably, several of these clonal groups have also been detected in remote wildlife reservoirs, underscoring their ecological versatility and global dissemination potential (Mora et al., 2018). The limited detection of canonical *pks* loci outside phylogroup B2 suggests that their long-term maintenance is favored within specific genomic backgrounds, although divergent or fragmented loci may be under-detected in draft assemblies.

The pronounced enrichment of *pks* in B2 lineages is consistent with a model in which canonical *pks* loci were acquired early during the diversification of some B2 lineages and subsequently maintained predominantly through vertical inheritance. The strong conservation of the *pks* locus across diverse strains further argues against frequent horizontal transfer, consistent with previous reports (Marcq et al., 2014; Putze et al., 2009). In addition to internal locus conservation, analysis of the chromosomal flanking regions demonstrated that the *pks* island occupies a recurrent genomic neighborhood across genomes, with a conserved upstream gene block and limited alternative flanking configurations. This pattern aligns with previous reports showing integration of the *pks* island near conserved asn tRNA-associated chromosomal loci. Such positional conservation supports limited chromosomal relocation and long-term stabilization within specific B2 lineages. The maintenance of the *pks* island is unlikely to be explained by a single factor. Instead, it probably reflects a complex interaction between lineage background, host-associated ecology, metabolic cost, niche persistence, bacterial competition, immune interactions, and compatibility with the broader accessory genome (Chagneau et al., 2022). Accordingly, reduced plasmid or AMR burden alone is unlikely to fully explain long-term pks persistence. External selective pressures may also contribute to *pks* persistence, including host-specific colonization niches, gut microbial competition, inflammatory microenvironments, and long-term adaptation to extraintestinal or intestinal reservoirs (Chagneau et al., 2022; Dalmasso et al., 2014). Our data do not establish the mechanistic basis of *pks* maintenance. However, the combination of conserved locus structure, restricted lineage distribution, and reduced accessory genome burden is consistent with a model in which *pks* persists in genomic backgrounds where its metabolic cost may be offset by ecological or host-associated benefits (Chagneau et al., 2022).

A central observation of this study is the pronounced depletion of ARGs in *pks*-positive genomes. Compared with *pks*-negative strains, *pks*-positive genomes exhibit a markedly reduced ARG burden, with most strains carrying zero or a single resistance determinant and rarely exceeding this range. This depletion affects ARGs not co-occurring with replicon markers as well as ARGs co-occurring with plasmid replicon markers, indicating a genome-wide resistance scarcity rather than a localized effect. Notably, a similar association between *pks* carriage and reduced AMR has been reported in smaller-scale studies of human intestinal isolates, supporting the generality of this pattern (Sarshar et al., 2017).

This resistance-depleted profile contrasts sharply with that of globally disseminated multidrug-resistant clones, such as ST131, which rely heavily on plasmid-mediated accumulation of resistance determinants to thrive in clinical environments (Mathers et al., 2015; Nicolas-Chanoine et al., 2014). The limited resistance repertoire observed here suggests that *pks*-positive genomes mark a lineage-restricted population in which reduced AMR burden is associated with clonal background, reduced plasmid replicon content, and conserved chromosomal architecture.

Consistent with their low resistance burden, *pks*-positive genomes display a strikingly plasmid-poor architecture. The near absence of plasmid replicons in the majority of *pks*-positive genomes indicates that plasmid acquisition and maintenance are less frequent in these lineages. Given that plasmids represent the principal vectors for horizontal ARG dissemination in *E. coli*, this structural feature may partially contribute to the low prevalence of plasmid-associated resistance observed in *pks*-positive populations. The reduced plasmid replicon burden observed in pks-positive genomes may reflect, among other possibilities, selective constraints associated with maintaining energetically demanding biosynthetic machinery. However, this remains a hypothesis rather than a demonstrated mechanism in the present study and requires experimental validation. Plasmids are known to impose fitness costs that can limit their persistence in the absence of strong selective pressure, particularly in genomes already carrying metabolically demanding traits (San Millan and MacLean, 2017). One possible explanation is that *pks*-positive lineages are adapted to ecological contexts in which host persistence, colonization efficiency, or microbial competition provide greater fitness benefits than plasmid-mediated resistance. In contrast, multidrug-resistant plasmid accumulation may be favored primarily under strong antibiotic selection in clinical environments (Dalmasso et al., 2014; Mathers et al., 2015; San Millan and MacLean, 2017).

Taken together, these results identify *pks*-positive *E. coli* as a genomically distinct subpopulation characterized by lineage restriction, low plasmid burden, and reduced resistance gene carriage. This constellation of traits suggests that *pks*-positive lineages may occupy ecological niches linked to long-term host association and persistence rather than short-term survival under intense antimicrobial pressure (Dalmasso et al., 2014).

The apparent mutual exclusivity between colibactin carriage and multidrug resistance raises important questions regarding evolutionary trade-offs in bacterial pathogenicity. Whether these traits are mechanistically incompatible or simply reflect adaptation to different ecological and clinical environments remains an open question that warrants further experimental investigation.

Several limitations should be acknowledged. This study relies on public genome data, so some assemblies are incomplete and metadata (host origin, geography) may be missing or imprecise—factors that could affect plasmid detection and annotation. Because pks detection relied on long nucleotide alignments to a canonical reference locus, fragmented or divergent *pks*-like islands, particularly outside phylogroup B2, may have been missed. Therefore, our conclusions primarily apply to high-confidence complete canonical pks loci rather than the full diversity of colibactin-like biosynthetic islands. Additionally, our findings are based on genomic associations; experimental validation is needed to confirm the mechanisms underlying the association between *pks* carriage, reduced plasmid burden, and lower AMR content.

In conclusion, our large-scale genomic analysis indicates that *pks* carriage in *E. coli* is not broadly distributed across the species but is concentrated within a limited number of clonal and phylogenetic backgrounds. *pks*-positive genomes were strongly associated with phylogroup B2 and characterized by low plasmid replicon burden and reduced AMR gene carriage. The strong conservation of the canonical *pks* locus across diverse lineages and chromosomal contexts is consistent with long-term vertical maintenance and contrasts with the more dynamic, plasmid-driven evolution observed in multidrug-resistant lineages. Together, these findings highlight divergence between chromosomally conserved virulence-associated secondary metabolism and resistance-driven accessory genome expansion. By defining the genomic context of colibactin production, this study provides a foundation for future work on how lineage-associated genomic strategies influence pathogenic potential, ecological fitness, and clinical outcomes in *E. coli*.

## Data Availability

The datasets presented in this study can be found in online repositories. The names of the repository/repositories and accession number(s) can be found in the article/Supplementary material.
