# Supplementary material for: Colibactin (pks) carriage in Escherichia coli is associated with lineage restriction, reduced plasmid burden, and lower antimicrobial resistance
Source: Front Microbiol. 2026 May 26;17:1842853. doi: 10.3389/fmicb.2026.1842853 (PMC13246691; doi:10.3389/fmicb.2026.1842853)
Supplement: Supplementary file 1 [file Supplementary_File.docx]

**Supplementary**


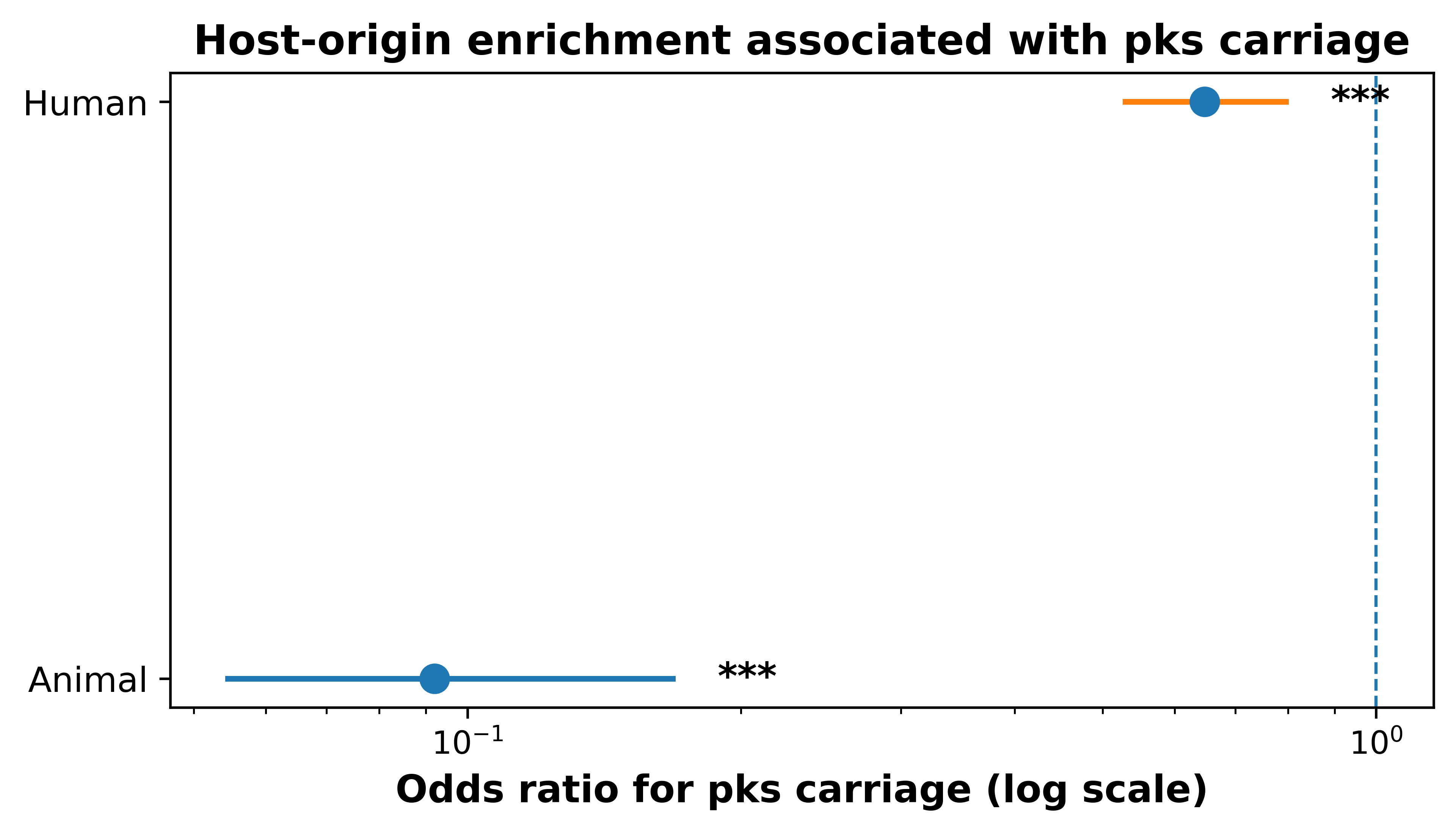


**Figure S1. Host-origin enrichment associated with canonical *pks* carriage**

Odds ratios were calculated relative to the full 9,700-genome dataset using genomes with available host metadata. Human-associated isolates represented the largest annotated category by raw counts but were not overrepresented after normalization, whereas animal-associated isolates were significantly depleted. Environmental isolates were not included due to very low sample size (n = 3). Genomes lacking host metadata were excluded from this visualization because “unknown” reflects missing metadata rather than a biological host category.


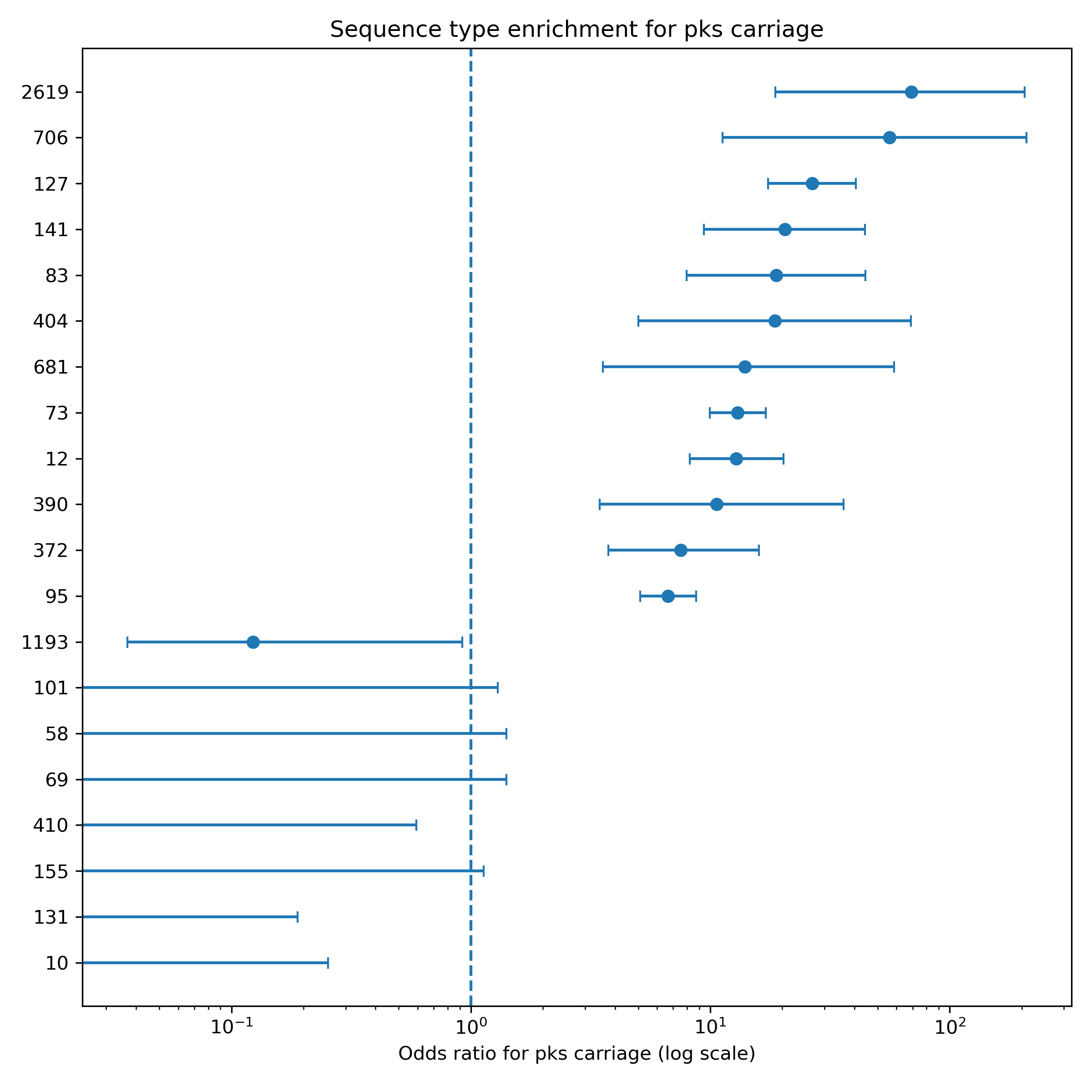


**Figure S2. Sequence-type enrichment associated with canonical pks carriage**

Forest plot showing odds ratios for association between major sequence types and canonical pks carriage relative to the full 9,700-genome dataset. Values >1 indicate enrichment among pks-positive genomes, whereas values <1 indicate depletion. Confidence intervals represent 95% exact confidence limits. Strong enrichment was observed for ST73, ST95, ST127, ST12, and several less frequent lineages, whereas multiple common non-pks lineages were depleted.


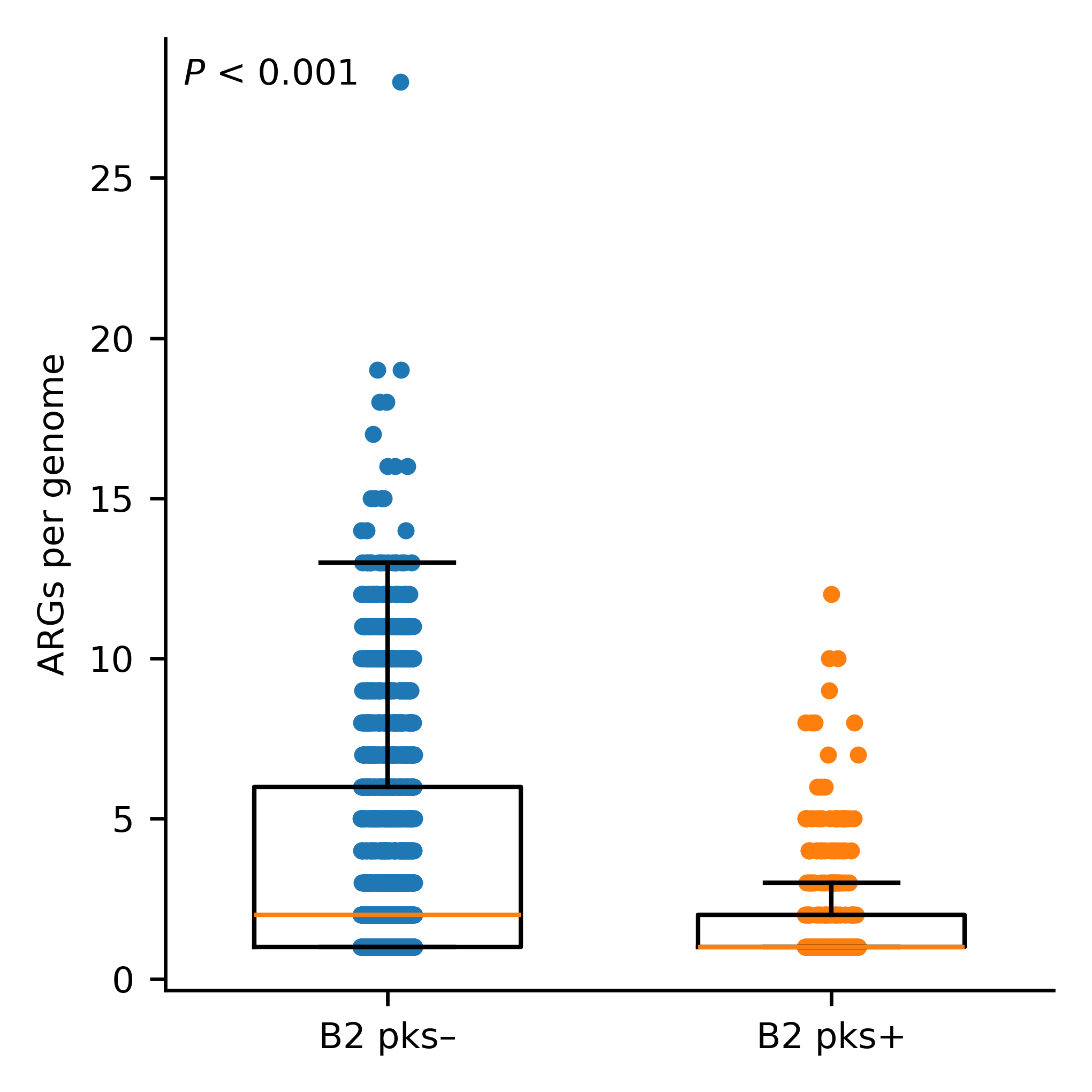


**Figure S3. Total ARG burden in phylogroup B2 genomes according to *pks* status**

Distribution of the total number of antibiotic resistance genes (ARGs) per genome among phylogroup B2 strains stratified by pks carriage. B2 *pks-*negative genomes exhibit a broad and right-skewed distribution of ARG counts, with multiple high-burden outliers, whereas B2 *pks-*positive genomes display a markedly constrained ARG profile with most genomes carrying ≤2 ARGs. The difference in ARG burden between B2 *pks-*positive and B2 *pks-*negative genomes is statistically significant (*Wilcoxon rank-sum test*, *P* < 0.001). (*pks+*: *pks-*positive, *pks-*: *pks-*negative)


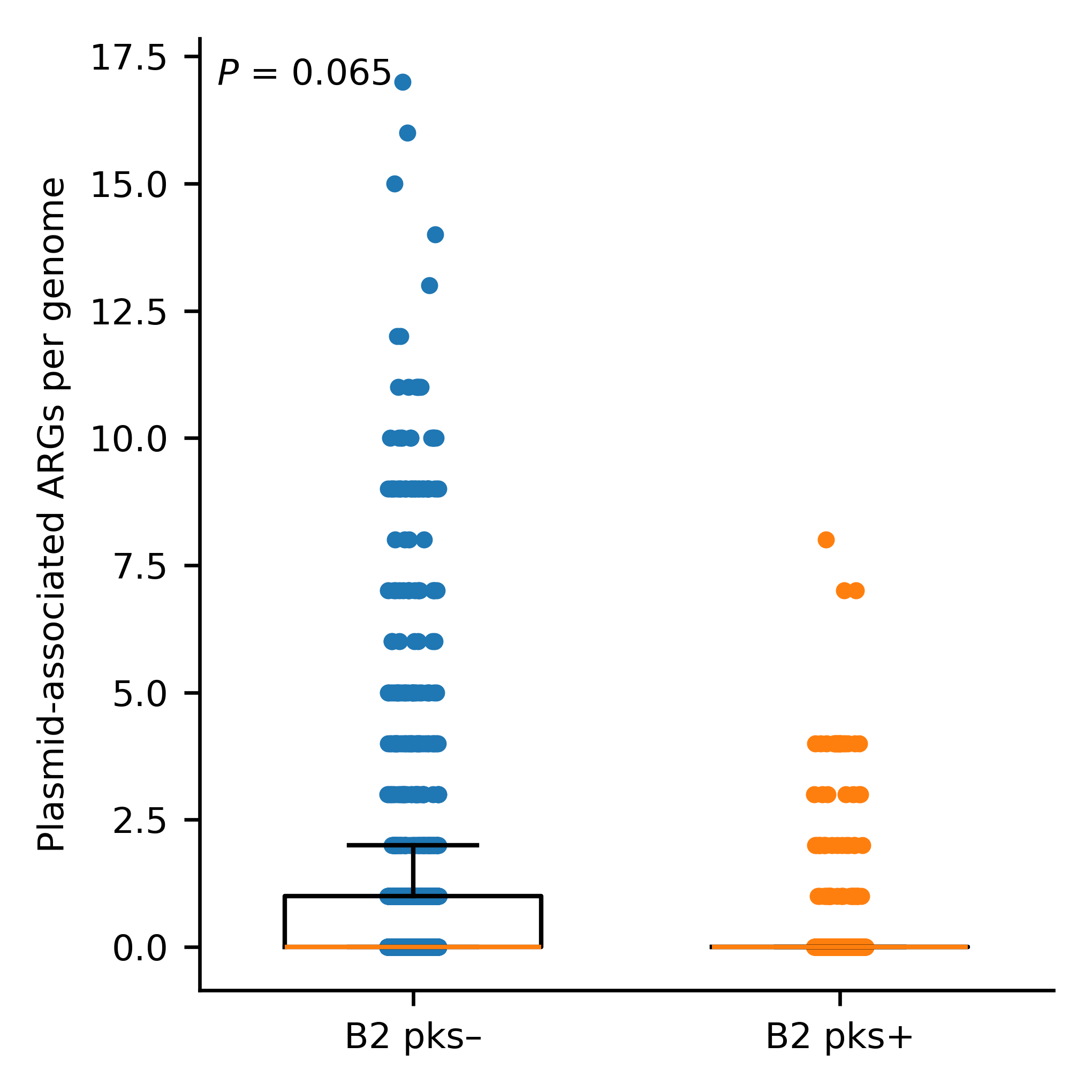


**Figure S4. Plasmid-associated ARG burden in phylogroup B2 genomes according to *pks* status**

Distribution of plasmid-localized ARGs per genome in B2 strains. B2 *pks-*negative genomes show a wide distribution of plasmid-associated resistance genes, with several genomes harboring multiple plasmid ARGs. In contrast, B2 *pks-*positive genomes are largely devoid of plasmid-borne resistance, with most genomes carrying zero plasmid ARGs. The difference approaches significance (*Wilcoxon rank-sum test*, *P* = 0.065), consistent with the strong depletion of plasmid carriage observed in the pks-positive population.





**Figure S5. Gene-level conservation of the *pks* genomic island across *pks*-positive *E. coli* genomes.**
Presence–absence heatmap of the 19 colibactin-associated genes (*clbA*–*clbS*) across a uniform subsample of 250 *pks*-positive *E. coli* genomes. Each row represents a genome and each column represents a *clb* gene. Gene presence is indicated by colored cells, while absence is shown in white. The heatmap reveals near-complete conservation of the *pks* gene repertoire, with only sporadic gene absences observed at low frequency and without clustering by phylogroup or sequence type.


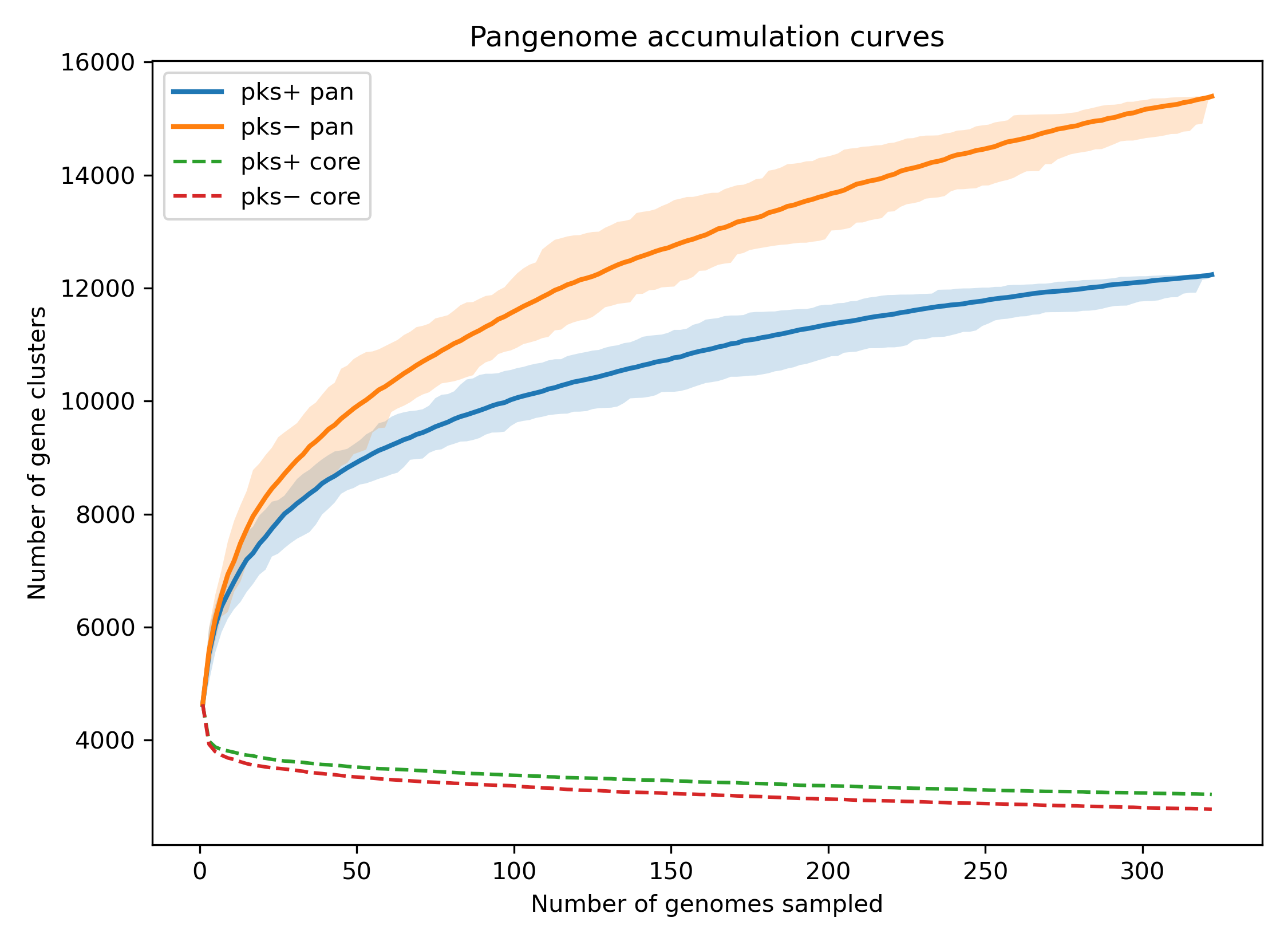


**Figure S6. Pangenome accumulation dynamics of B2-matched *pks*-positive and *pks*-negative *E. coli* genomes.**
Pangenome (solid lines) and core genome (dashed lines) accumulation curves reconstructed from randomly sampled genomes using Panaroo. Shaded areas represent variability across permutations. The *pks*-negative group (orange) shows a faster and continuous expansion of the pangenome, indicating greater gene repertoire diversity and higher genomic plasticity. In contrast, the *pks*-positive group (blue) displays a slower pangenome growth and maintains a larger core genome (green dashed), reflecting increased genomic conservation and structural stability. These patterns support the existence of a genomically constrained *pks*-positive subpopulation compared to the more flexible *pks*-negative B2 genomes.


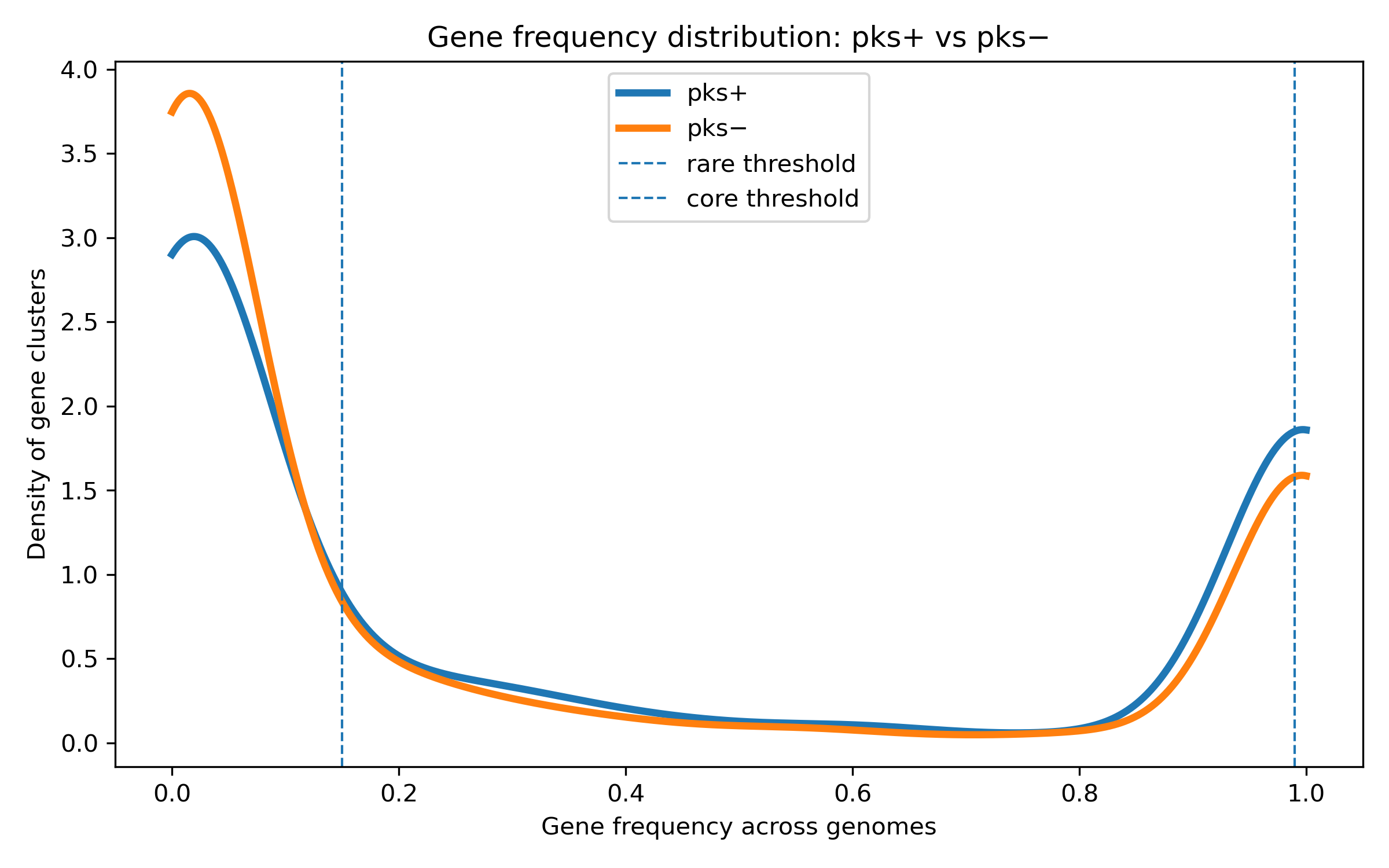


**Figure S7. Gene frequency spectrum in B2-matched *pks*-positive and *pks*-negative *E. coli* genomes.**
Density distribution of gene cluster frequencies across genomes for *pks*-positive (blue) and *pks*-negative (orange) populations. Vertical dashed lines indicate thresholds defining rare genes (left) and core genes (right). The *pks*-negative group shows a higher density of low-frequency genes, consistent with an expanded accessory genome and increased genomic plasticity. In contrast, *pks*-positive genomes exhibit a stronger enrichment of high-frequency (core) genes, reflecting a more conserved genomic architecture. This distribution further supports the existence of a genomically stabilized *pks*-positive subpopulation compared to the more variable *pks*-negative B2 lineages.


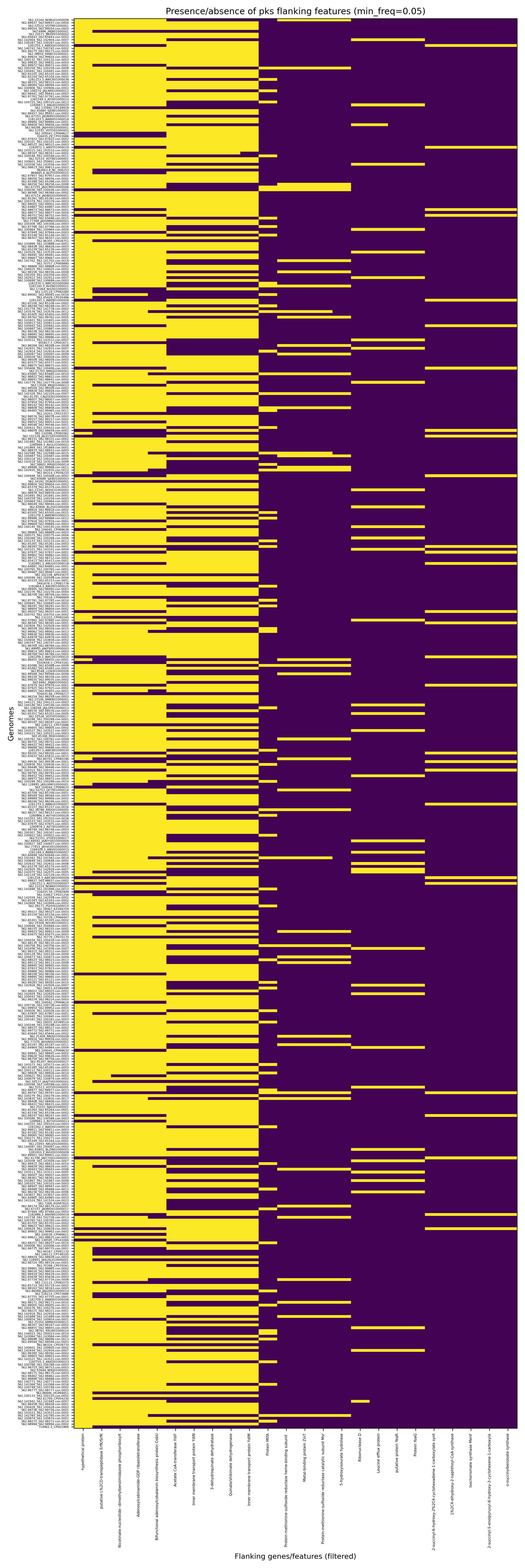


**Figure S8. Positional conservation of the chromosomal neighborhood flanking the *pks* genomic island.**

Binary presence/absence heatmaps of genes located in the immediate chromosomal vicinity of the *pks* locus across *pks*-positive *E. coli* genomes. Flanking regions were defined as annotated genes directly upstream and downstream of the *pks* cluster in each genome assembly. Only features detected in ≥5% of genomes were retained for visualization. Yellow indicates presence and purple absence. A highly conserved block of upstream flanking genes is evident across the majority of genomes, whereas downstream regions display comparatively greater variability but still contain recurrent conserved elements. The predominance of a shared chromosomal neighborhood and the absence of alternative flanking gene configurations support positional stability of the *pks* insertion site, consistent with long-term chromosomal integration rather than frequent independent insertion events.
